# ABodyBuilder: Automated antibody structure prediction with data–driven accuracy estimation

**DOI:** 10.1080/19420862.2016.1205773

**Published:** 2016-07-08

**Authors:** Jinwoo Leem, James Dunbar, Guy Georges, Jiye Shi, Charlotte M. Deane

**Affiliations:** a Department of Statistics, University of Oxford, Oxford, UK; b Roche Pharma Research and Early Development, Large Molecule Research, Roche Innovation Center Munich, Penzberg, Germany; c Informatics Department, UCB Pharma, Slough, UK

**Keywords:** Antibody modeling, model quality assessment, modeling, nanobody, protein structure prediction

## Abstract

Computational modeling of antibody structures plays a critical role in therapeutic antibody design. Several antibody modeling pipelines exist, but no freely available methods currently model nanobodies, provide estimates of expected model accuracy, or highlight potential issues with the antibody's experimental development. Here, we describe our automated antibody modeling pipeline, ABodyBuilder, designed to overcome these issues. The algorithm itself follows the standard 4 steps of template selection, orientation prediction, complementarity-determining region (CDR) loop modeling, and side chain prediction. ABodyBuilder then annotates the ‘confidence’ of the model as a probability that a component of the antibody (e.g., CDRL3 loop) will be modeled within a root–mean square deviation threshold. It also flags structural motifs on the model that are known to cause issues during in vitro development. ABodyBuilder was tested on 4 separate datasets, including the 11 antibodies from the Antibody Modeling Assessment–II competition. ABodyBuilder builds models that are of similar quality to other methodologies, with sub–Angstrom predictions for the ‘canonical’ CDR loops. Its ability to model nanobodies, and rapidly generate models (∼30 seconds per model) widens its potential usage. ABodyBuilder can also help users in decision–making for the development of novel antibodies because it provides model confidence and potential sequence liabilities. ABodyBuilder is freely available at http://opig.stats.ox.ac.uk/webapps/abodybuilder.

## Abbreviations

Fv: Variable Fragment
CDR: Complementarity–Determining Region
AMA: Antibody Modeling Assessment
RMSD: Root–Mean Square Deviation
NGS: Next–Generation Sequencing
ESSS: Environment–Specific Substitution Score

## Introduction

Antibodies are an important class of biotherapeutics.^1^ They effectively bind their cognate antigens with high specificity and high affinity.^2^ In humans, antigen binding occurs on the variable fragment (Fv) between the variable domains of the heavy and light chains (VH and VL, respectively). These two domains contain 6 complementarity–determining region (CDR) loops, which form the majority of the antigen binding site.^2,3^ Three of these loops, CDRH1, CDRH2, and CDRH3, are from the VH domain, and 3, CDRL1, CDRL2, and CDRL3, are from the VL domain. Other organisms, such as camelids, have single–domain antibodies that are composed of only the heavy chain; such nanobodies have only the 3 heavy chain CDR loops.^4^ The CDR loops' relative positions and their amino acid sequences determine most of an antibody's binding properties. Other structural features, such as the relative orientation of the antibody's domains,^5^ are known to affect an antibody's affinity. Thus, knowledge of an antibody's structure is useful for clinical and biotechnological applications.^6,7^

The immense diversity of antibodies,^8-10^ and the value of structural models in engineering antibodies,^6,11^ have motivated the development of several computational antibody modeling tools.^7,11-15^ To compare such methodologies, 2 Antibody Modeling Assessments have been held.^6,16^ In the second competition (AMA–II), 7 methods were benchmarked on their ability to model 11 Fvs;^6,17^ only 3 of these methods (RosettaAntibody, PIGS, and Kotai Antibody Builder)^18-20^ are freely available. The competition was divided into 2 stages: modeling the entire Fv (stage I), or only the CDRH3 loop, given the crystal structure of the remaining Fv (stage II). In stage I, most Fvs were modeled relatively well (average root mean square deviation [RMSD] of the Fv backbone: 1.1Å), though the CDRL1, CDRL3, CDRH1, and the CDRH3 loops were often modeled with lower accuracy.^6,17^

Currently, most antibody modeling pipelines follow a 4–stage workflow, with minor variations in the steps. Initially, a template structure is chosen for the target antibody, either for the VH and VL domains separately, or for both domains combined.^7,12-14^ Alternatively, a fragment-based method can be used to assemble the VH and VL domains.^15^ The VH–VL orientation^21^ is then modeled after choosing the framework template.^22^ In the third stage, the ‘canonical’ CDR loops (CDRH1, CDRH2, CDRL1, CDRL2, CDRL3) are modeled, followed by CDRH3. The models may also be refined.^18^

No current pipelines comment on the expected accuracy of the model, so a model's accuracy is only known *post hoc*, once a crystal structure has been determined. From a user's perspective, it is impossible to determine how useful a prediction will be. This is particularly problematic for the CDRH3 loop, which can be modeled extremely poorly (RMSD >5Å) in some targets.^6^ In addition, no current publicly available pipeline comments on the in vitro ‘developability’ of the antibody.^23,24^ Antibodies are prone to several post–translational modifications that hinder the production and retention of the antibody. More importantly, these modifications can adversely affect the antibody's functional properties.^23,24^ Thus, knowledge of these sequence liabilities prior to experimental work could reduce the rate of failure of producing functional antibodies.

Here, we present ABodyBuilder, an antibody modeling pipeline that uses our increasing knowledge–base of antibody structures^10^ to guide decision–making in modeling antibodies. The overall methodology behind ABodyBuilder is similar to other pipelines. We select template structures based on sequence identity, and, if necessary, predict the antibody's orientation based on the ABangle parameters.^21^ All 6 CDR loops are then predicted by FREAD^25-27^ using a CDR–specific database. The model's side chains are then completed by SCWRL4.^28^

ABodyBuilder differs from other methods in that it annotates the confidence of a model as the probability that a region (e.g., the framework) will be modeled within a specific RMSD threshold. ABodyBuilder also flags structural motifs within the model antibody that are known to hinder *in vitro* development.^23^ Finally, ABodyBuilder is the only publically available software that is capable of modeling nanobodies (e.g., camelid VHH antibodies). Unlike other pipelines that allow manual input,^18-20^ ABodyBuilder is a rapid, fully automated method for antibody model generation, making it ideal for challenges such as modeling large, next–generation sequencing (NGS) data sets.^29-31^ Here, we show that ABodyBuilder produces models of similar quality to other leading methods in its fully automated mode, and describe how it provides meaningful information for antibody development.

## Results

### Framework selection

**Figure 1.**
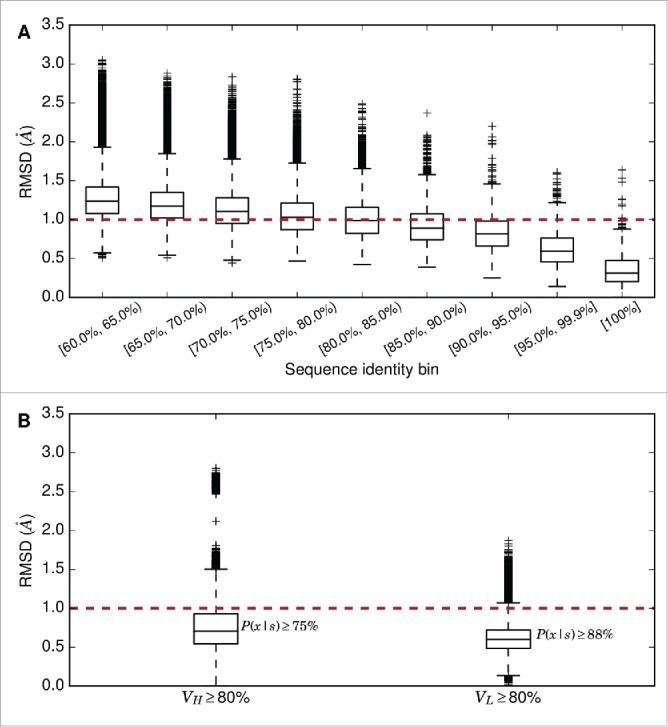
(A) Boxplot of pairwise Fv–Fv framework region superimpositions in the redundant set; only pairs with sequence identity ≥60 % are shown. (B) Boxplot of pairwise VH – VH framework region superimpositions and VL–VL framework region superimpositions where sequence identity ≥80 %.

The first stage in ABodyBuilder is the selection of a single template, or 2 templates (one for the VH and one for the VL), to model the framework region. In order to determine how sequence identity between template and target influences the accuracy of model building, the framework regions of all pairs of structures in our redundant set were superimposed. First, both chains were superimposed (Fv–Fv superimposition), and second, the heavy and light chains were superimposed separately (VH–VH or VL–VL). The RMSD between the pairs were compared to their sequence identities (Fig. 1).

Given our observations, we use a single ‘global’ template (both VH/VL structures and orientation) if a single template structure for the target could be found with ≥80% sequence identity for both heavy and light chains' framework regions. In this scenario, we expect to have a sub–Angstrom template for the VH and VL domains with a probability of 0.75. If either chain has <80% sequence identity to the target, 2 separate structures are used, and the orientation of the highest sequence identity global template is used (example template selections are described in Table S1).

### Modeling the CDR loops

**Figure 2.**
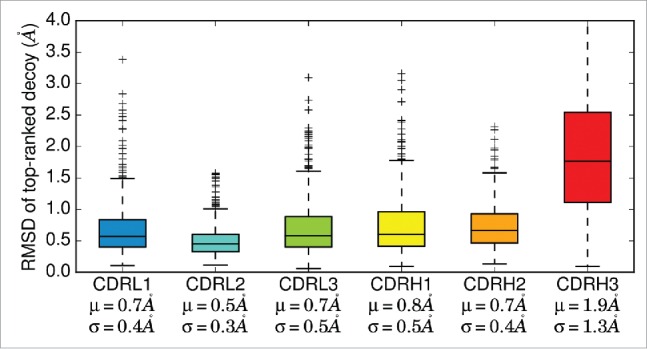
RMSD distributions of the top–ranked decoy from FREAD for each CDR loop. FREAD was used to model individual CDR loops on template framework structures of our non–redundant set. The RMSD was calculated by superimposing the backbone atoms of the framework regions of the template and target. Decoys with RMSD >4 Å are not displayed. μ: mean; σ: standard deviation.

Once a template framework structure is selected, ABodyBuilder uses FREAD,^25-27^ a database method, to model the CDR loops. A CDR–specific database was used for each CDR loop; if a suitable decoy was not found in the database, an Fv–specific database was used. If a decoy is still not found, the most sequence–similar, length–matched CDR loop (based on its BLOSUM62 score) is used as the template. If no length–matched templates are found, the most sequence–similar loop is then used as the template for *ab initio* modeling by MODELLER (see Methods).^32^ Fig. 2 shows the accuracy of individual CDR loop predictions from FREAD on template framework structures for our non–redundant set. In this initial assessment, the RMSD between the model and native CDR loops was calculated after superimposing both chains' framework regions' backbone atoms (i.e., excluding the CDR loops). CDRL2 was modeled with the highest accuracy (average backbone RMSD 0.5Å), followed by CDRL1, CDRL3, CDRH2, CDRH1. CDRH3 was modeled with the lowest accuracy (average backbone RMSD 1.9Å).

The order of CDR loop modeling is important because each modeled CDR may influence the conformations of the next CDR loop. We used the accuracy of predicting individual CDR loops and the occurrence of Cβ−Cβ contacts between CDR loops (Figs. S2, S3) to decide the ordering. The CDR loops are modeled in the following order: CDRL2, CDRH2, CDRL1, CDRH1, CDRL3, and CDRH3. The CDRL2 loop is modeled first as it is usually predicted with the highest accuracy. Next, CDRH2 is modeled as it is the best predicted CDR loop within the heavy chain, and is not in contact with CDRL2. CDRL1 and CDRH1 follow on as they are the next–best predicted CDR loops on the light and heavy chains, respectively. Finally, the CDRL3 is modeled before CDRH3. An alternative order of CDRL2, CDRL1, CDRL3, CDRH2, CDRH1 and CDRH3 was considered on the basis of FREAD accuracy per variable domain. As the results were unaffected, the proposed order was retained. When modeling a nanobody, the order is conserved, i.e., CDRH2, CDRH1, CDRH3.

### Side chain modeling

**Figure 3.**
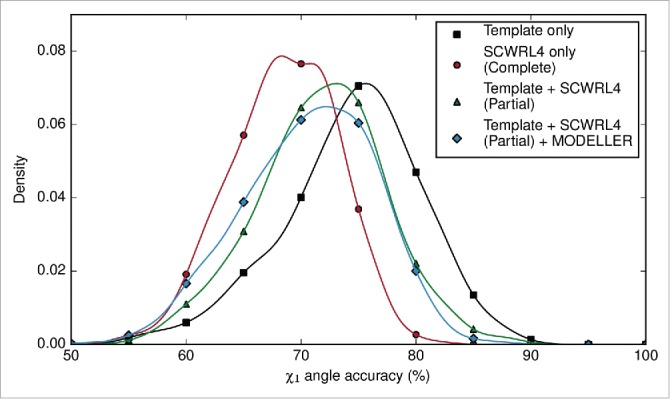
Density plot of χ_1_ angle accuracy (%) for side chain prediction, using only the template's rotamers, completely re–modeling every side chain using SCWRL4, or using both the template rotamers where available, and SCWRL4s rotamers elsewhere. The χ_1_ angle accuracy of models that were refined by MODELLER is also shown. Note that the χ_1_ angle accuracy for ‘Template Only’ is calculated from fewer rotamer comparisons as it is based on comparing only the identical residues between the template and target.

At this stage in the ABodyBuilder methodology, we have a complete backbone, and side chains where the template and target share identical residues. The side chains of the target antibody could be modeled by 2 different methods. ‘Complete’ prediction, where every side chain is predicted, or alternatively, ‘partial’ prediction where side chains of identical residues from the template are retained, and the remaining side chains are predicted. The side chains of the framework region and the CDR loops were either completely re–modeled or partially predicted using SCWRL4.^28^ Side chain clashes occasionally arise from SCWRL4 predictions. We removed these by subjecting the clashing side chains to an initial round of relaxation by MODELLER.^32^ If clashes are still found in the structure, all side chains are relaxed. The χ_1_ angle and χ_1+2_ angle accuracies of the complete and partial predictions were compared. Our analyses showed that preserving side chains of common residues leads to better accuracy than re–modeling every side chain (Fig. 3); however, MODELLER relaxation decreases the χ_1_ angle accuracy at the expense of resolving clashes in the model structure.

### Confidence estimates

**Figure 4.**
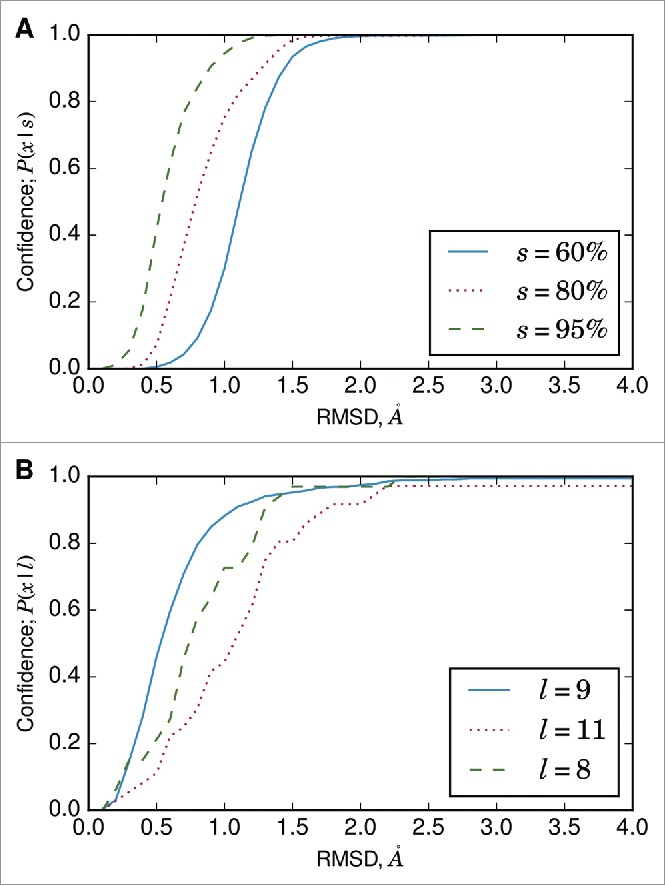
(A) Conditional probability (Equation 1) curve for VH framework region accuracy. Framework superimposition data on the redundant set (Fig. 1) was used to calculate the probability that a framework region will be modeled within *x*Å. The calculations depend on the sequence identity of the template. (B) Conditional probability curve for CDRL3 loop accuracy. The calculations depend on the length of the CDRL3 loop, and the 3 most common CDRL3 loop lengths (as defined by North *et al.*^*33*^) are shown.

ABodyBuilder estimates the confidence of the model antibody structure as the probability that a region (e.g., framework, CDRL3) will be modeled within *x*Å given the sequence identity or loop length (Fig. 4). Thus, the confidence calculations can also be used to obtain the expected RMSD for a specified probability. The confidence measures are data–driven, and are based on the results from the pairwise framework region superimpositions or the FREAD predictions for the individual CDR loops. They are empirical approximations, and are not *de facto* RMSD values for the model.

We use our ∼1.2 million pairwise superimpositions of framework regions (Fig. 1) to estimate these confidence values. For example, a template VH domain with 80% sequence identity to a target VH domain will be modeled at RMSD ≤1.0Å with a probability of 0.75. This does not necessarily mean that the VH domain will have RMSD ≤1.0Å to the target. Rather, it states that there is a 75% chance that the target will be modeled at RMSD ≤1.0Å. Confidence increases with the RMSD threshold, i.e., there is a greater probability that a region will be modeled with lower accuracy, or higher RMSD.

We also estimate the accuracy of the CDR loops as a function of loop length. The backbone RMSDs of the top–ranked FREAD predictions on template framework regions for our non–redundant set were used to estimate the confidence of modeling a CDR loop.

### Benchmarking ABodyBuilder

To benchmark ABodyBuilder, it was tested on the antibodies in the non–redundant set, and on our blind test set, which is a set of 136 structures that have been deposited in SAbDab since we built our methodology. When modeling these structures, sequence–identical antibodies were ignored. The accuracy of the models was calculated as described in the Methods section. Briefly, the Fv and framework regions' RMSD was calculated after superimposing the backbone atoms for both chains (Fv RMSD), or for each chain separately (framework region RMSD). For the CDR loops, the backbone atoms of each chain's framework region were superimposed, and the RMSD between the model and native CDR loops was calculated thereafter, similar to the method used in the AMA–II competition.^6^ Our RMSD calculations use North *et al.*'s CDR loop definitions, whereas the AMA–II competition calculated a model's RMSD using Chothia's definitions.^6,33^ As discussed later, when using the AMA–II dataset, we used the Chothia definition to be consistent with the competition.^3,6^

ABodyBuilder gives confidence estimates in its structural predictions. A complete antibody is modeled as 8 regions (2 framework regions and 6 CDR loops), and each of these is separately considered. Comparably, a nanobody's 4 regions (one framework and 3 CDR loops) are annotated individually. In all our models, a default confidence of 75% was used to calculate the expected RMSD. This value indicates, based on our framework region superimpositions and FREAD results on individual CDR loops, that there is a 75% chance that a component will be modeled within *x*Å. These confidence measures were used to identify components that would be difficult to model.

ABodyBuilder modeled the 462 ‘complete’ Fvs in the non–redundant set with average backbone RMSD for the Fv of 1.3Å. On average, the canonical CDR loops were predicted with sub–Angstrom accuracy (average backbone RMSD 0.5Å, 0.4Å, 0.6Å for CDRL1, CDRL2, and CDRL3, respectively, and 0.6Å, 0.6Å, 1.9Å for CDRH1, CDRH2, and CDRH3, respectively). The RMSD values are lower than those in the original investigation using FREAD to predict CDR loops.^27^ It appears that the increase in available structural data has led to an improvement in CDR loop modeling. Sixty of the 79 nanobodies in the non–redundant set were VHH antibodies, with an average domain RMSD of 2.6Å; the average backbone RMSD for the CDRH1, CDRH2, and CDRH3 loops were 1.4Å, 0.9Å and 3.5Å, respectively. On the other hand, the domain RMSD of the 19 VL–only nanobodies was 1.0Å; the average backbone RMSD of the CDRL1, CDRL2, and CDR3 loops were 0.6Å and 0.3Å, and 1.1Å respectively.

The confidence metric was particularly useful in identifying CDR loops that were modeled poorly. For instance, the CDRH3 loop of 2vxv:HL was estimated with 75% confidence to be modeled within 3.1Å, and its actual RMSD was 3.0Å. However, the default confidence measure can over– or under–estimate accuracy. For example, ABodyBuilder was 75% confident that the CDRH1 loop of 3aaz:HL is modeled within 1.3Å, although its actual RMSD was 1.7Å. The framework regions' confidence measure is relatively robust, but, in the case of the CDR loops, the lack of data can lead to less accurate confidence estimates.

ABodyBuilder was also used to build models of 136 structures (108 Fvs, 24 VHHs, 4 VL–only antibodies) that were deposited in the PDB between 24 February 2015 and 20 December 2015 (our blind test set). Here, the average backbone Fv RMSD of ‘complete’ antibodies was 1.5Å. Similar to the non–redundant set, the ‘canonical’ CDR loops of Fvs were predicted with sub–Angstrom accuracy (average backbone RMSD 0.6Å, 0.5Å, and 0.8Å for CDRL1, CDRL2 and CDRL3, respectively; 0.6Å and 0.7Å for CDRH1 and CDRH2, respectively; Fig. S4). For the 24 VHH antibodies, the domain RMSD was 1.9Å, and the CDRH1 and CDRH2 loops were predicted with average backbone RMSDs of 1.5Å and 1.1Å (Fig. S4). In contrast, the VL–only antibodies were predicted with sub–Angstrom accuracy for the entire domain (0.6Å), and for the CDR loops (0.4Å, 0.2Å and 0.6Å for CDRL1, CDRL2, and CDRL3; Fig. S4). Despite the low averages, the blind test set posed several challenges. Some canonical CDR loops were poorly modeled (e.g., the CDRL1, CDRL2, and CDRL3 loops of one target, 4yfl:HL), and *ab initio* modeling was necessary for modeling the CDRL3 loop of 5c0n:CD. The average RMSD for the CDRH3 loops in the blind test set was 2.1Å for Fv antibodies, and 2.4Å for VHH antibodies. Fifty–nine of the CDRH3 loops in this set were long (≥15 residues), which may explain the high average backbone RMSD; furthermore, 51 of the CDRH3 loops were not modeled by FREAD (50 were modeled by a sequence similar template, one *ab initio*). For CDRH3 loops not modeled by FREAD, it was not possible to determine the confidence, but we suggest that they are likely to be modeled poorly. Overall, the results on these 2 datasets suggest that ABodyBuilder can generate high–quality models for most targets.

### Benchmark on AMA–II targets

ABodyBuilder was also tested on the 11 antibodies from the AMA–II competition.^6,17^ To replicate the blind test conditions as far as possible, all structures that were deposited in the PDB after 31 March 2013 were omitted from the template search and FREAD databases. The accuracy of the models was calculated as described in the Methods section, and here the Chothia–defined CDR loops were used as in the AMA–II competition.^6^

The ABodyBuilder models were of similar quality to that of the methods used in AMA–II (Fig. S5). The average RMSD for the whole Fv for our models was 1.2Å; this is comparable to other publically available pipelines: RosettaAntibody (1.1Å), Kotai Antibody Builder (1.1Å), and PIGS (1.5Å). Except for the CDRL2 loop where the average backbone RMSDs of ABodyBuilder models (0.3Å) were marginally lower (RosettaAntibody: 0.4Å, Kotai Antibody Builder: 0.3Å, PIGS: 0.5Å), the average backbone RMSD of all other CDR loops was far lower. In particular, ABodyBuilder showed an improvement of >0.5Å RMSD for the CDRL3 and CDRH3 loops compared to the other pipelines. This is likely due to the choices made by FREAD. For Ab06 (PDB: 4m6o), ABodyBuilder selected 3hr5:HL as the template framework, as done by the Schrodinger group;^14^ however, ABodyBuilder used 1om3 over 2aab as its CDRH3 template. Despite the differences in environment–specific substitution scores (ESSS; 1om3: 26, 2aab: 47), FREAD's ranking based on anchor RMSD (1om3: 0.188Å vs. 2aab: 0.223Å) led to this selection, which was ultimately a better template for CDRH3. In many cases, ABodyBuilder generated the top, or joint–top prediction for a component of an antibody. However, some cases were more challenging to ABodyBuilder in comparison to other pipelines, such as the CDRL1 loop of Ab05 (PDB: 4m6m, RMSD 2.8Å). Here, ABodyBuilder chose 1lgv because it had the lowest anchor RMSD (0.127Å), despite its low ESSS (31). Of the top 10 predictions, 3h42 had the highest ESSS (96) but was ranked fourth in terms of anchor RMSD (0.151Å); using this template would have led to a prediction with a backbone RMSD of 0.8Å.

### Large–scale modeling of antibody sequences

Given the growing availability of large datasets of antibody sequences,^31^ in particular from NGS,^29,30^ a desirable aspect of an antibody modeling pipeline is the ability to rapidly generate models. To test the scalability of ABodyBuilder, a non–redundant set of 6267 (3490 human, 2373 mouse) paired antibody sequences from DIGIT,^34^ Abysis (http://www.abysis.org), and SAbDab^10^ were modeled. For any sequence that required *ab initio* intervention by MODELLER,^32^ only one model was generated.

**Figure 5.**
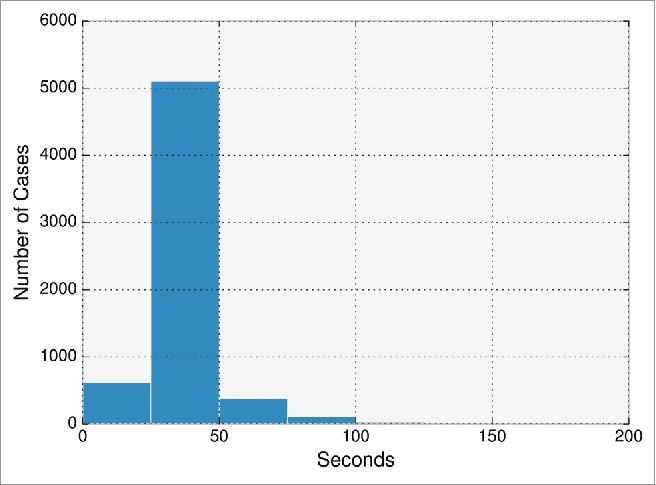
Time elapsed by ABodyBuilder in modeling the non-redundant set of 6267 paired antibody sequences from DIGIT,^34^ Abysis (http://www.abysis.org.) and SAbDab.^10^ Time is measured from when the sequence is given as input to ABodyBuilder until it finishes re–numbering models into the IMGT numbering scheme. For each target requiring *ab initio* modeling by MODELLER (e.g., CDR loops), only one possible model was generated.

The average runtime for each sequence was 34 seconds, taking 222.9 seconds at most. In total, the entire set of 6267 paired sequences was modeled in 3552 CPU hours (Fig. 5), which is ∼567 CPU hours per 1000 sequences. This compares to ∼250,000 CPU hours per 1000 sequences that were modeled in a recent study modeling antibodies from an NGS data set of human antibody sequences.^30^ In this study, the framework region RMSD to a non–redundant set of crystal structures was 1.0 ± 0.29Å. The CDR loops' RMSDs were only calculated for models of naïve antibodies, ranging from 0.8–2.4Å.^30^ In contrast, for every ABodyBuilder model, model confidence is annotated where possible. At the default 75% confidence level, the average expected RMSD values are 0.9Å and 0.7Å for the heavy and light chain framework regions, and 0.8Å, 0.7Å, 1.0Å, 1.1Å, 1.0Å, and 3.1Å for the CDRL1, CDRL2, CDRL3, CDRH1, CDRH2, and CDRH3 loops, respectively. The models are currently hosted in http://opig.stats.ox.ac.uk/webapps/abodybuilder/models, and the time elapsed for each model is shown.

### Server output

As described above, ABodyBuilder can rapidly build accurate models of antibodies from sequence. Once a model has been generated, it can be downloaded by the user, or interactively analyzed. We created a web server application for ABodyBuilder, which is freely available at http://opig.stats.ox.ac.uk/webapps/abodybuilder.

For each model structure, an annotations page is created using PV (Fig. S6).^35^ Here, the secondary structure elements, domains, solvent exposure, confidence measures, and the sequence liabilities can be visualized on the model structure. For example, if a particular target antibody has an N–linked glycosylation motif^36^ in its CDRL1 sequence (Asn–X–Ser/Thr), this portion of the CDRL1 is then highlighted if its relative accessible surface area is >10%.^37^ Each sequence liability is given a unique color, allowing simultaneous visualization of multiple liabilities (should they be present). A full list of annotated sequence liabilities is provided in Table S2.

## Discussion

Here, we describe ABodyBuilder, an antibody modeling pipeline that builds antibody models that are comparable to other current freely available methodologies, but is the first to offer model accuracy estimates. ABodyBuilder can also model complete Fvs, or nanobodies. ABodyBuilder follows the archetypal 4–stage workflow that is characteristic of most antibody modeling pipelines. In order to design ABodyBuilder, we tested several strategies for every step and selected the best method given the currently available structural data.

The framework region's RMSD was rarely above 1.5Å in all the models generated in our study. The framework region had the highest average RMSD after CDRH3; this may be due to the length dependence of RMSD. However, the relatively high RMSD value may also stem from 2 features: VH/VL orientation, and the framework loops. Currently, ABodyBuilder re–orients the VH and VL chains using the ABangle parameters from the best available global template. However, predictions from machine learning methods^22^ or energy minimization^12,38^ could be used to enhance prediction in the future. The choice of the template could also be enhanced by the use of detailed structural features, for example, ‘bulge’ structures near the N–terminus of the VH domain.^39^

ABodyBuilder models the ‘canonical’ CDR loops with sub–Angstrom accuracy (on average) in the blind test and AMA–II datasets. In the AMA–II set, the CDRL1 and CDRL3 loops were modeled particularly well in comparison to the other methods (Fig. S5), reinforcing the benefits of a knowledge–based approach.^6^ However, FREAD was incapable of generating a decoy in some cases (e.g., CDRL3 of 5c0n:CD). This is perhaps due to the lack of data in FREAD's databases; for example, 5c0n:CD represents a rabbit antibody. In 31 March 2013 (the deposition date cut–off for benchmarking with AMA–II targets), there were only 6 rabbit antibody structures, and as of 27 January 2016, there are only 11 in SAbDab.^10,40^ However, as FREAD's performance has already improved since the previous benchmark,^27^ and the amount of antibody data in the PDB steadily increases, we expect predictions to continue to improve over time.

The choice of a poor framework template could have affected the prediction of the CDRL1 loop of Ab05. The template's light chain framework region had a backbone RMSD of 1.5Å. Furthermore, the target structure has an unusual tilt angle, as previously commented;^15^ our chosen template had a high deviation in tilt angle with respect to the target structure. Both features could have affected the choice of the CDRL1 loop by FREAD.

Once ABodyBuilder generates a model structure, it displays several features of the antibody model, including the confidence measurements and sequence liabilities. Both metrics are intended to assist users in pursuing *in vitro* development of their target antibody. The confidence measures successfully identified structures that were modeled poorly. This feature of ABodyBuilder will help users to identify cases where current methodologies may not be able to produce high–quality decoys. However, the confidence calculations assume that the distribution of antibody sequences in SAbDab is representative of all antibody sequences. Therefore, if a query sequence is very different from those in SAbDab, the assumptions underlying our confidence measures will not hold, and incorrect estimates may occur.

ABodyBuilder currently identifies 11 possible sequence liabilities that are known to affect antibody development (Table S2). In our non–redundant set, ABodyBuilder identified an N–linked glycosylation site in the VH framework region of cetuximab (PDB: 1yy8, IMGT position H97),^41^ and aspartate isomerization sites in the CDRL1 of omalizumab (PDB: 2xa8, IMGT positions L34, L36).^42^ However, these liabilities were identified solely on the basis of the sequence motif and solvent accessibility. Thus, ABodyBuilder will flag sequence motifs as liabilities despite the fact that they may only be problematic in certain conditions (e.g., low pH).

ABodyBuilder automatically builds high–quality models for most targets in ∼30 seconds, allowing users to quickly obtain a model structure for any antibody sequence. Our tool uniquely estimates the model's confidence and flags sequence liabilities; in particular, we demonstrate that the confidence estimate can help identify components that have been poorly modeled. ABodyBuilder serves to facilitate antibody design by translating candidate antibody sequences into structural prototypes for further study, such as antibody–antigen docking and antibody humanization.

ABodyBuilder is freely available as a web application at http://opig.stats.ox.ac.uk/webapps/abodybuilder.

## Methods

All of the antibody sequences to be modeled by ABodyBuilder were numbered using the IMGT numbering scheme^43^ via ANARCI,^44^ and the CDR loop positions were those defined by North *et al.*^*33*^ At the end of the modeling process, ABodyBuilder annotates model structures with all the major numbering schemes and CDR definitions (e.g., Chothia;^3^ Kabat^45^). For all prediction steps, sequence identical antibodies were ignored.

### Datasets

Our initial dataset was a redundant set of antibodies with resolution ≤2.5Å, downloaded from SAbDab on 24 February, 2015. Structures that could not be numbered, or those with unusual structures (e.g., PDB: 1oay, PDB: 1sjv; Fig. S1) were omitted, leaving 1170 structures (998 complete Fvs, 1104 VHs and 1064 VLs). For benchmarking FREAD and the rotamer modeling method, a non–redundant set of 541 antibodies was used (462 complete Fvs, 79 nanobodies; 522 VHs and 481 VLs overall), based on a 90% Fv sequence identity cutoff using CD–HIT.^46^ A ‘blind test’ set of 136 structures (with resolution ≤4.0Å; 108 complete Fvs and 28 nanobodies) that had been deposited in the PDB between 24 February 2015 and 20 December 2015 was used to blind test the ABodyBuilder pipeline. To test the efficiency of ABodyBuilder, ABodyBuilder was run on a non–redundant set of 6267 paired antibody sequences from DIGIT,^34^ Abysis (http://www.abysis.org/), and SAbDab^10^ based on a 99% Fv sequence identity cutoff.

### Calculation of model accuracy

For all measurements of accuracy, we represent RMSD as the backbone RMSD between the model and native structures. Fv RMSD is calculated by superimposing all backbone atoms between the model and native structures. Similarly, the framework regions' RMSD is determined for each chain of the model by superimposing the framework backbone atoms to the corresponding chain in the native structure.

For each of the CDR loops, accuracy is calculated by first superimposing the respective chain's framework region backbone atoms, then calculating the RMSD between the loops, similar to the method used in the AMA–II competition.^6^ The only difference is in the definition of the CDR loops; we use North *et al.*'s definition rather than the Chothia–defined CDR loops.^6,33^ However, for calculating the CDR loops' RMSD for the AMA–II targets, the Chothia–defined CDR loops were used. To measure the accuracy of the CDRH3 loop, the heavy chains' framework regions are first superimposed, and the RMSD is then calculated between the model and native CDRH3 loops. Likewise, for the CDRL3 loop, the light chains' framework regions are superimposed before calculating the RMSD between the CDRL3 loops. In our initial analysis when we determined the order of CDR loop modeling, the RMSD between CDR loops was calculated after superimposing the backbone atoms of both chains' framework regions, which is a more stringent test.

### Template selection

Because ABodyBuilder is a homology modeling pipeline, the first step is template selection. In order to identify templates, ABodyBuilder searches SAbDab for structures with a resolution of 2.5Å or better that are close in sequence to the target. Template selection is based on sequence identity over the framework region of the Fv, i.e., residues that are outside of North *et al.*'s^33^ CDR definitions. ABodyBuilder can either select a single ‘global’ template from one antibody (VH/VL framework structures and the orientation) or a ‘hybrid’ template, where 2 template structures, one for the VH and one for the VL framework, are used.

### VH–VL orientation prediction

If a ‘global’ template is selected, the VH–VL orientation is given by that template. For hybrid templates, the VH and VL domains are re–oriented using the orientation from the highest sequence identity global template. This re–orientation procedure is carried out as described in Bujotzek *et al.*^*22*^ Briefly, the Cα coordinates of the ABangle consensus structure are transformed according to the ABangle parameters from the global template. The heavy and light chains of the hybrid template are then superimposed to the rotated consensus structure.

### CDR prediction

The CDR loops are modeled sequentially in an order determined by our ability to accurately predict them, and the contacts between them. The CDR loops are predicted by FREAD, which is a database search algorithm that selects for potential structures based on anchor Cα separations, its ESSS, and anchor RMSD.^25-27^ For each CDR loop, FREAD first searches for fragments from a CDR–specific database. Each of the six CDR–specific databases contains a particular CDR loop's fragments; in other words, a CDRL1 database only contains CDRL1 loop fragments. The six CDR–specific databases were built using the redundant data set to capture various conformations of sequence–identical loops.

If a suitable decoy cannot be found for a given CDR loop, a second iteration of FREAD is performed on the missing CDR loop(s) using an Fv–specific database. The Fv–specific database contains all possible fragments of antibodies from the redundant dataset. If FREAD fails to find a decoy from the CDR–specific or Fv–specific databases, the most sequence–similar, length–matched CDR loop with resolution ≤2.5Å is used as the template. Sequence similarity is determined by using the BLOSUM62 score between the template and target CDR loops. If there are no length–matched templates, an *ab initio* prediction is performed using MODELLER.^32^

Of the 3009 CDR loops in the non–redundant set, 164 loops were predicted by using the most sequence–similar loop, and 17 required *ab initio* modeling. For our blind test set of 136 antibodies, 732 CDR loops were predicted, of which 74 were predicted using the most sequence–similar loop, and 10 were modeled *ab initio*. In the AMA–II set, only one of the 66 was modeled by using the most sequence–similar loop. Finally, for the 37602 CDR loops from the set of 6267 paired antibody sequences from DIGIT,^34^ Abysis (http://www.abysis.org/), and SAbDab,^10^ 2166 were predicted by using a sequence–similar template, and 230 were modeled *ab initio*.

### Side chain prediction

SCWRL4^28^ is used to predict the side chain rotamers of residues that are not identical between the template and target. The model is then checked for backbone and side chain clashes; 2 atoms are considered to be clashing if the distance between them is less than 65% of the sum of their van der Waal's radii. For example, the van der Waal's radius of a carbon atom is 1.7Å; thus, 2 carbon atoms would be clashing if they are less than 2.21Å apart. If clashes are detected, models are first relaxed by MODELLER^32^ by only refining the clashing residues. If clashes still exist, then all side chains are refined by MODELLER.^32^ Rotamer accuracies were calculated as the fraction of rotamers that were ‘correct’, i.e., the fraction of rotamers within 40° of the native rotamer.^28^

### Confidence measurements

The confidence we have in a segment of the model is calculated for a specific RMSD threshold as a conditional probability, given the sequence identity of that segment. If the segment is a CDR loop, the loop length is used. Confidence measurements for the framework region are based on the pairwise superimpositions carried out on the redundant set. The probability that the framework region will be modeled with ≤*x*Å accuracy for a sequence identity bin *s* is calculated as

(1) $$P(x|s)=\frac{P(x\cap s)}{P(s)}$$

and each bin is 1% wide. To obtain the probability of a sequence identity bin, *P*(*s*), we first calculate the sequence identity of antibodies *a* and *b, S(a,b)*, in the set of all antibodies, *A*. We then divided the pairs of antibodies with sequence identity *s*±2.5% by the number of all possible antibody pairs, i.e.,

(2) $$P\left(s\right)=\frac{\sum_{a\in A}\sum_{b\neq a,b\in A}I(S(a,b)\in s\pm 2.5)}{\left|A\right|(\left|A\right|-1)}$$

where |*A*| represents the cardinality of the set *A*, and *I* is the indicator function. The joint probability, *P*(*x*∩*s*), represents the probability that antibody pairs with sequence identity *s* ± 2.5% will have RMSD ≤*x*Å. Thus,

(3) $$P\left(x\cap s\right)=\frac{\sum_{a\in A}\sum_{b\neq a,b\in A}I(S(a,b)\in s\pm 2.5,RMSD(a,b)\leq x)}{\left|A\right|\left(\left|A\right|-1\right)}$$

For example, of the 608856 VH–VH superimpositions in the redundant set, 32904 are within sequence identity bin *s* = 80%. Thus,

$$P\left(80\right)=\frac{32904}{608856}\approx 0.0540.$$

Of the 32904 superimpositions within this sequence identity bin, 24696 superimpositions had RMSD ≤1 .0Å. Thus, the joint probability is calculated as

$$P\left(1.\text{0}\cap 80\right)=\frac{24696}{608856}\approx 0.0406.$$

Thus, the conditional probability that a template framework region would have ≤1.0Å RMSD to a target antibody, given 80% sequence identity, is

$$P(1.0|80)=\frac{0.0406}{0.0540}=0.752.$$

The framework regions' confidence measures are separate for VH and VL because we do not have a method that will reliably estimate the accuracy of orientation prediction.^21,47^ The CDR loops' confidence measures were based on the results from running FREAD on model framework structures of the non–redundant set. The conditional probability is calculated as a function of CDR loop length, *l*. As RMSD is dependent on length, it is possible to encounter situations where the CDR loops have higher confidence values at lower RMSD thresholds than the framework. For example, it is possible to have 75% confidence that CDRL1 is accurate to within 1Å, and 75% confidence that the framework is accurate to within 2Å. Furthermore, the lack of data per loop length bin for the CDR loops in comparison to a sequence identity bin for the framework may lead to poorer estimates of accuracy for the CDR loops.

The probability–based metric was preferred over estimating an RMSD value with error ε (i.e., estimating that a region will be modeled at *x* ± εÅ) as the latter assumes that each sequence identity or length bin has the same distribution of RMSD values. Our data showed that this is not the case (Fig. 1), and any measurement of ε (e.g., standard deviation) will vary for each bin. Thus, we opted for a distribution–free metric. Furthermore, the probability estimate allows a more dynamic expectation of a model's accuracy, as we can derive the accuracy for a wide range of RMSD thresholds (*x*) or probabilities (P(x|s)), depending on the user's application.

### Sequence liabilities

The target antibody's sequence and structure is analyzed for potential issues that can conflict with in vitro development. This is based on data collected from publications.^23,24^ Currently, ABodyBuilder flags 11 possible issues with antibody development; a full list is given in Table S2. For a predicted sequence liability, it is only visualized if the position's relative accessible surface area, calculated by DSSP,^37^ is greater than 10%.

## Supplementary Material

Supplementary_Figures_and_Tables.pdf
