# Supplementary material for: ABodyBuilder: Automated antibody structure prediction with data–driven accuracy estimation
Source: MAbs. 2016 Jul 8;8(7):1259–68. doi: 10.1080/19420862.2016.1205773 (PMC5058620; doi:10.1080/19420862.2016.1205773)
Supplement: Supplementary_Figures_and_Tables.pdf [file kmab-08-07-1205773-s001.pdf]

Table S1: Two example cases of template selection. In the case of 1h0d:BA, a global template is used as both chains of 1ejo:HL have ≥80% sequence identity to the target. In the case of 12e8:HL, a 'hybrid' template is used, as 1i3g:H has a sequence identity of 79.8%, and thus a second structure is used to predict the VH domain. However, 1i3g:HL had the highest global sequence identity (87%) and is thus used for re–orientation of the two chains.

Target Antibody	VH template (% Identity)	VL template (% Identity)	Orientation Template
1h0d:BA	1ejo:H (89%)	1ejo:L (86%)	1ejo:HL
12e8:HL	3nig:E (90%)	1i3g:L (95%)	1i3g:HL

Table S2: List of sequence liabilities and their motifs that are highlighted by ABodyBuilder.

Liability	Motif
Unpaired cysteines[1]	Free cysteines
N–linked glycosylation[2]	Asn-X-Ser/Thr (X not Pro)
Methionine oxidation[3]	Free methionines
Tryptophan oxidation[3]	Free tryptophans
Asparagine deamidation[4]	Asn-Gly/Ser/Thr
Aspartate isomerisation[4]	Asp-Gly/Ser/Thr/Asp/His
Lysine glycation[3]	Lys-Glu/Asp/Lys
N–terminal glutamate[5]	N–terminal glutamate
Integrin binding[6]	Arg-Gly-Asp, Arg-Tyr-Asp,
CD11c/CD18 binding[6]	Gly-Pro-Arg
Fragmentation[7]	Asp-Pro

Table S3: Choice of templates by ABodyBuilder in modelling antibodies from the AMA–II competition.

Antibody	Framework Template	Sequence Identity	Orientation Template	CDR Loop Templates					
				H1	H2	H3	L1	L2	L3
Ab01 (4ma3)	4jo2I-4jo2M	82.0	4jo2IM	3vfgH	2dquH	2pcpD	4jo2M	4jo2M	4jo2M
Ab02 (4kuz)	2w9dH-3mbxL	95.5	3o2dHL	2w9dH	2w9dH	1pg7Z	3mbxL	3mbxL	3hi5L
Ab03 (4kq3)	3macH-3eo9L	99.0	2cmrHL	3macH	3macH	1pg7I	3eo9L	3eo9L	3qpxL
Ab04 (4kq4)	3mxwH-3mxwL	85.0	3mxwHL	3mxwH	3mxwH	2g60H	3mxwL	3mxwL	3mxwL
Ab05 (4m6m)	2xwtA-2xwtB	90.5	2xwtAB	2xwtA	2xwtA	1wejH	1lgvA	2xwtB	2xwtB
Ab06 (4m6o)	3hr5H-3hr5L	93.0	3hr5HL	3hr5H	3hr5H	1om3K	3difC	3sgdI	3e8uL
Ab07 (4mau)	1f58H-1f58L	92.0	1f58HL	1f58H	1f58H	1fl5B	1f58L	1f58L	1f58L
Ab08 (4m7k)	1d5iH-2ap2A	95.0	1ap2BA	1d5iH	1d5iH	1xf3B	2ap2A	2ap2A	2ap2A
Ab09 (4kmt)	3nabH-3nabL	99.0	3nabHL	3nabH	1i3gH	3o2vH	3nabL	3nabL	2fr4A
Ab10 (4m61)	1kb5H-3ijhC	95.0	3ujtHL	1kb5H	1kb5H	1svzB	3ijhC	3ijhC	1orsA
Ab11 (4m43)	2w9dH-4gw5C	91.0	4dgiHL	2w9dH	4h0gA	4h0hB	4gw5C	4gw5C	4gw5C

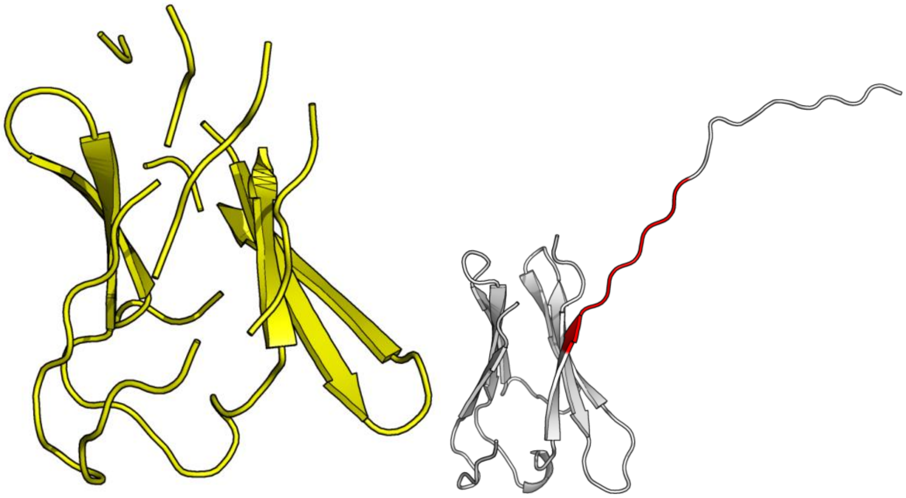

Figure S1: Examples of unusual structures (PDB: 1oay, 1sjv). Some of the chains are missing atom coordinates (left, yellow), or in the case of 1sjv (right, white), the framework region following CDRH3 (red) trails off.

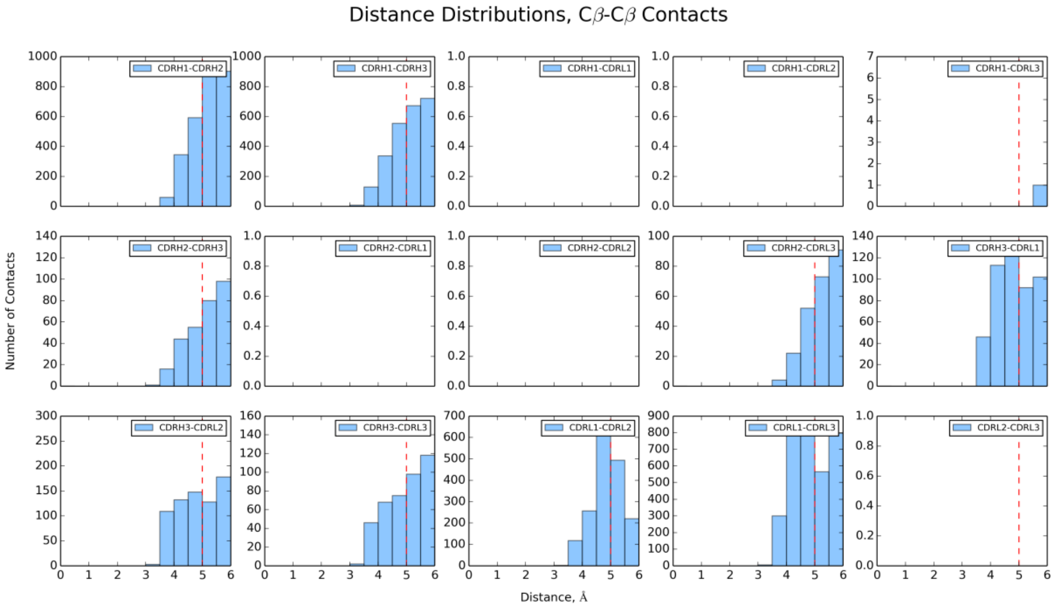

Figure S2: Histogram of Cβ–Cβ contacts between CDR loops of antibodies in the non–redundant set. The number of contacts within 5Å (red dotted line), and the accuracy of modelling CDR loops (Figure 2) were used to determine the order of CDR loop modelling.

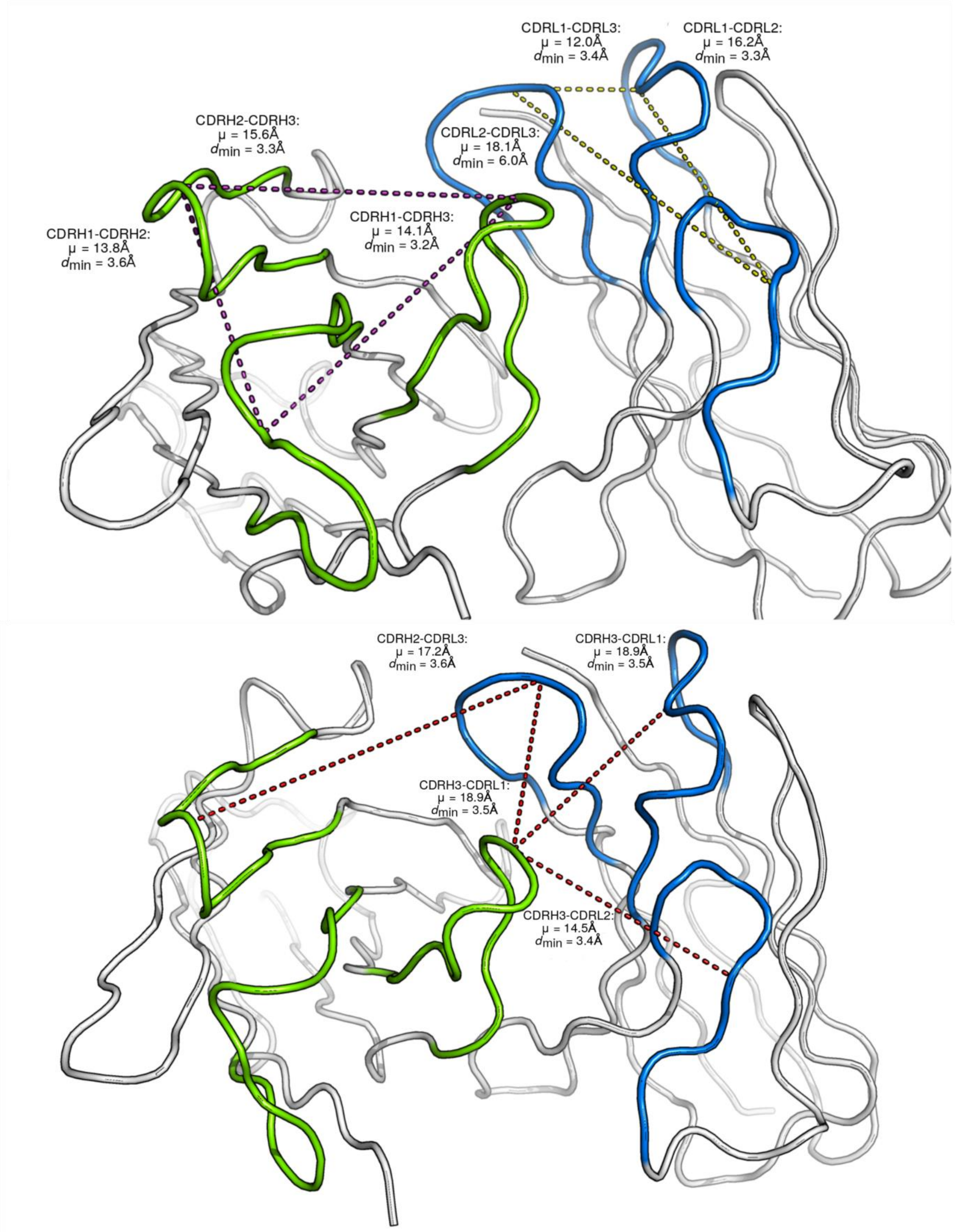

Figure S3: Mean and minimum Cβ–Cβ distances between CDR loops. If a pair of CDR loops' minimum Cβ–Cβ distance is >5Å (Figure S2), it is not shown. *Top*: Cβ–Cβ contacts between CDR loops within each variable domain. *Bottom*: Cβ–Cβ contacts between CDR loops between variable domains.

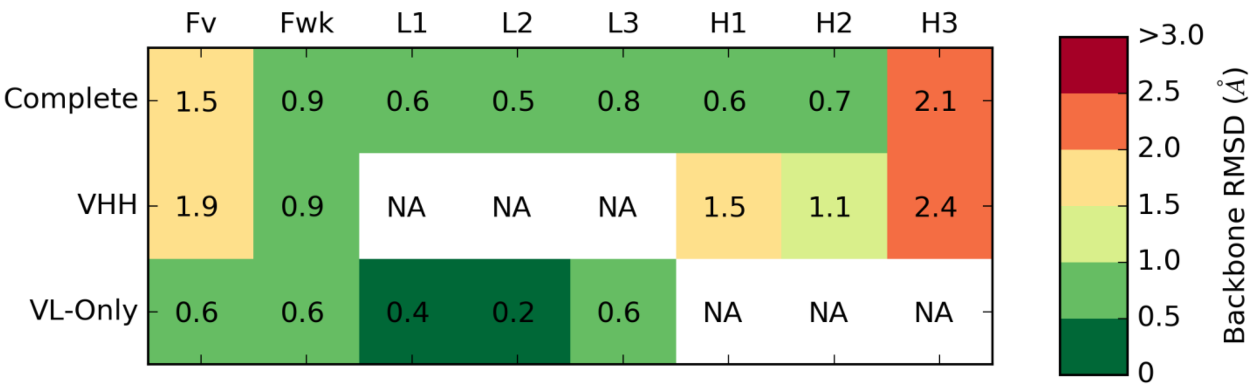

Figure S4: Heatmap of average backbone RMSD in the blind test set of 136 antibodies, divided into either complete Fvs, VHH, or VL–only antibodies. The RMSD of each component was calculated as described;[8] North *et al.*'s CDR definitions were used.[8, 9]

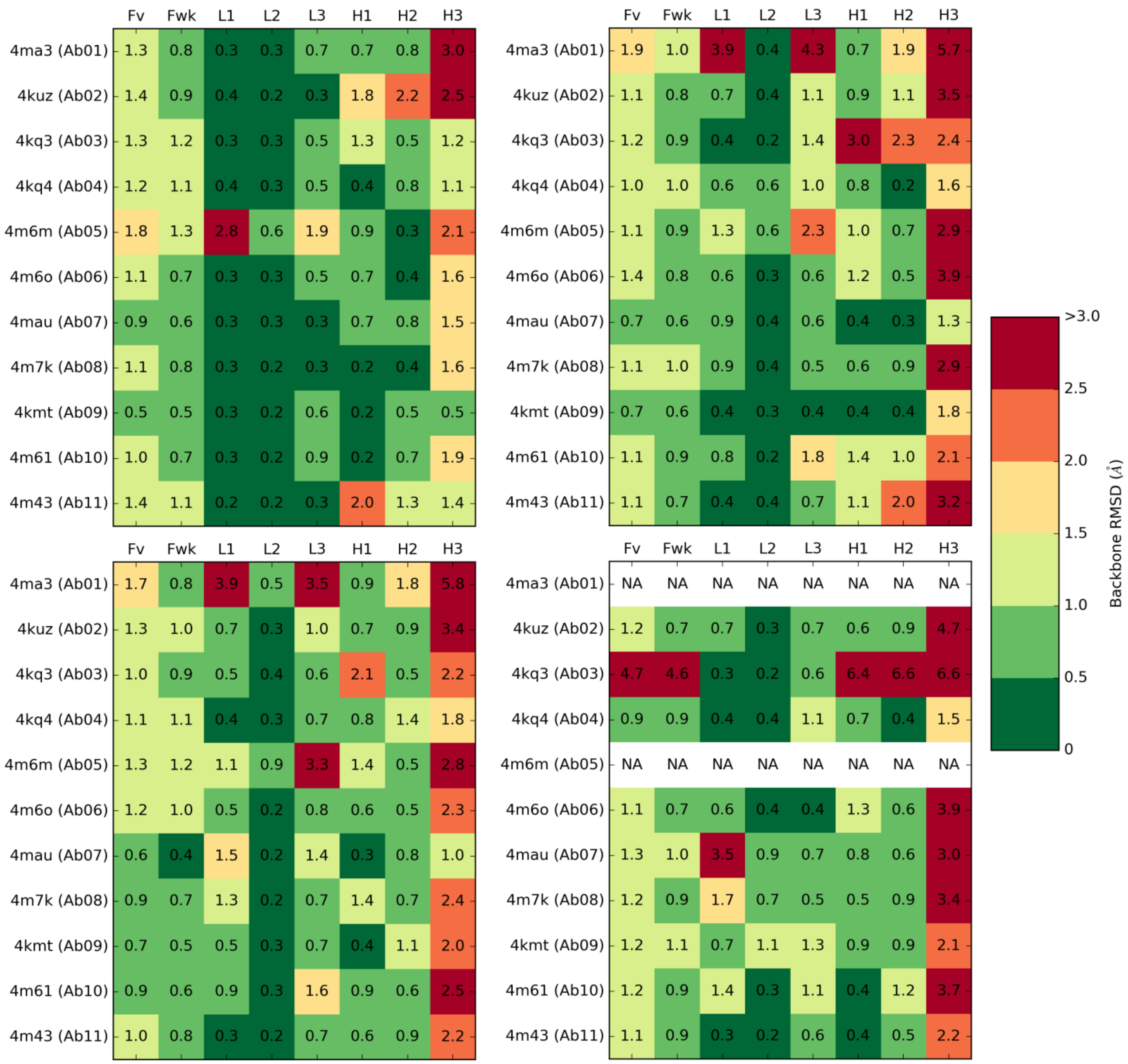

Figure S5: Backbone RMSD heatmap of different methods from the AMA–II competition, including ABodyBuilder (top–left), Kotai Antibody Builder[10] (bottom–left), RosettaAntibody[11] (top–right), and PIGS[12] (bottom–right). The RMSD of each region was calculated as described.[8] ABodyBuilder was run using only structures that were deposited in the PDB by 31 March, 2013, and the templates for each component are described in Table S3.

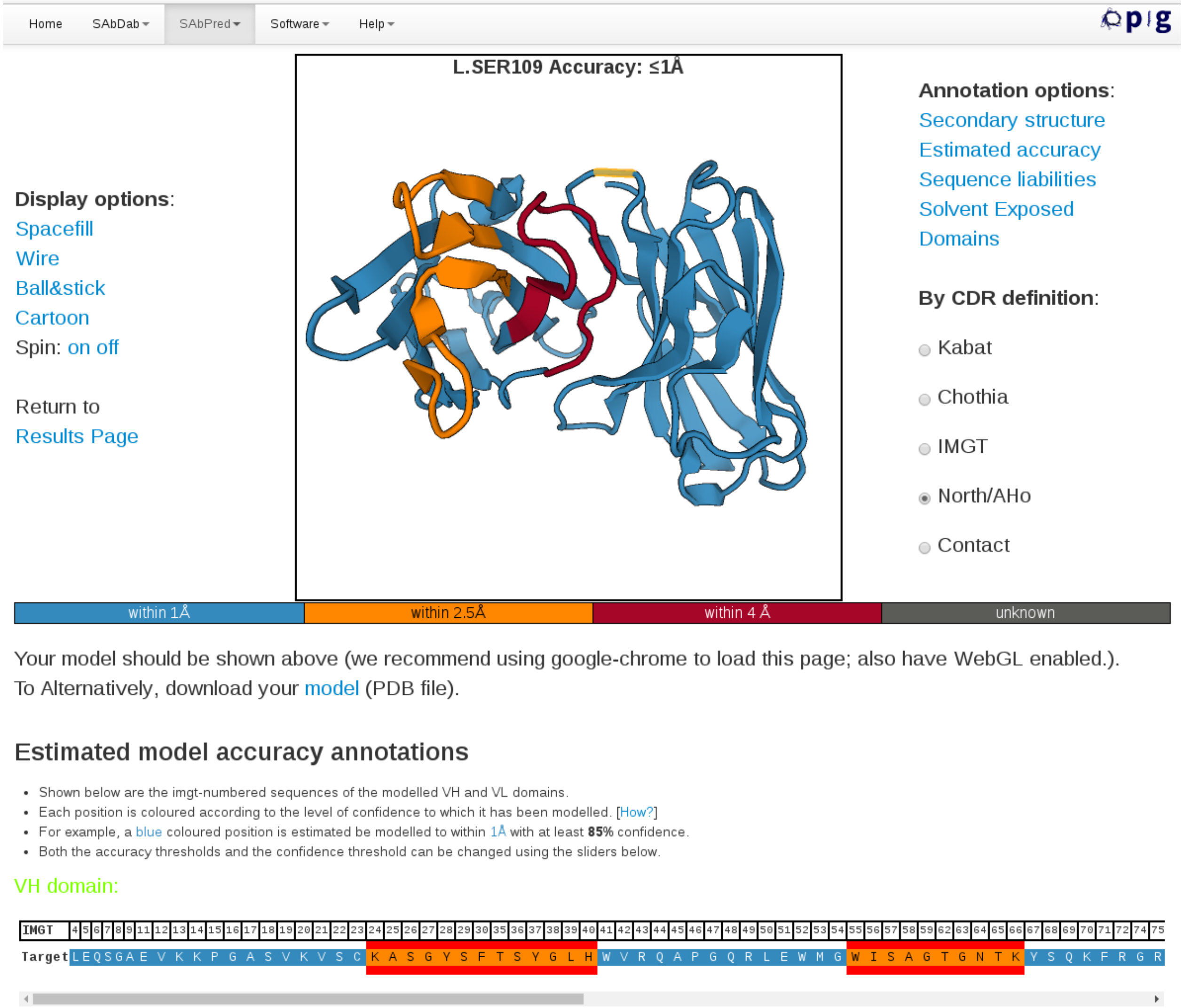

Figure S6: Screenshot of an example ABodyBuilder output (annotations page), generated using PV.[13] Users have the freedom to visualise specific features and download the model and accompanying data.

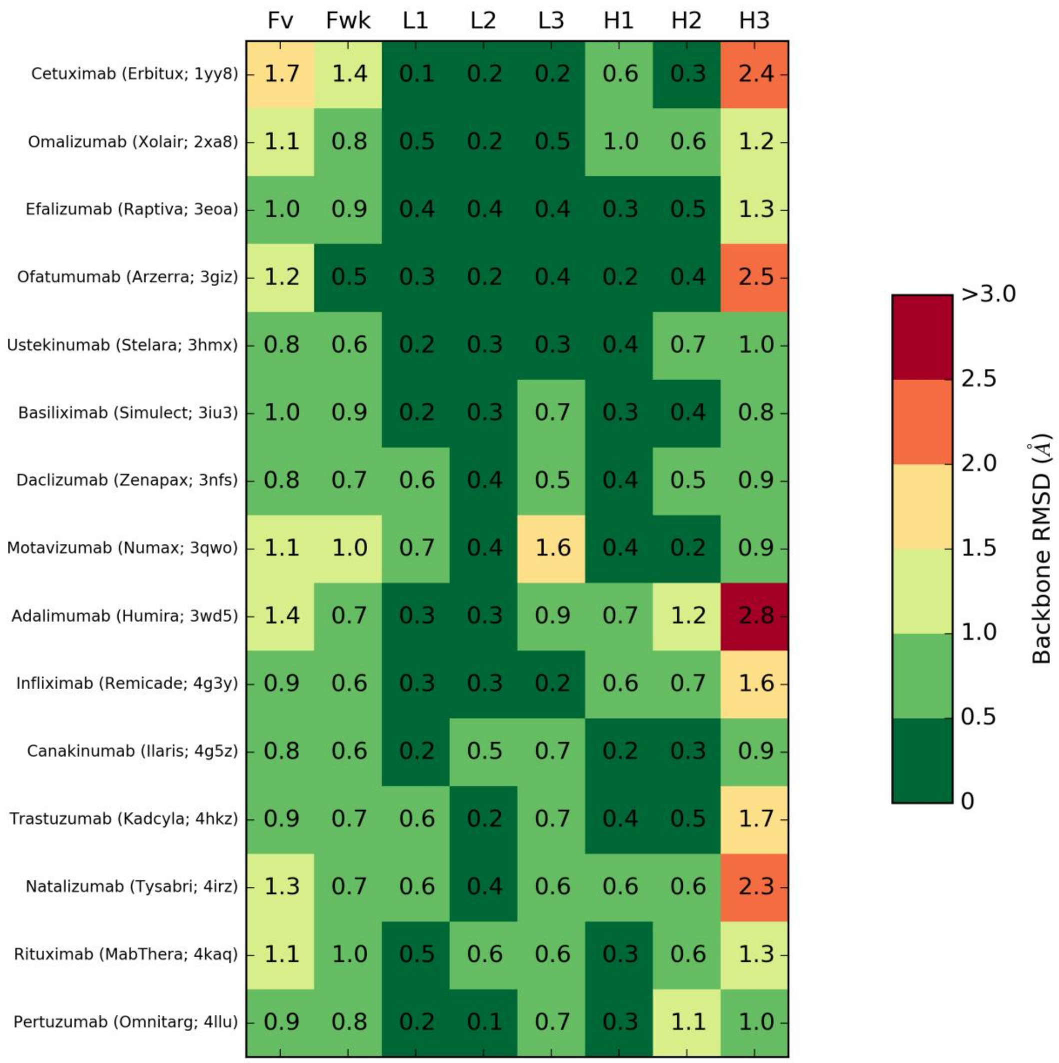

Figure S7: Backbone RMSD heatmap of therapeutic antibodies that were modelled by ABodyBuilder. For each antibody, its trade name and PDB code are provided.

Supplementary References

1. Brych SR, Gokarn YR, Hultgen H, Stevenson RJ, Rajan R, Matsumura M. Characterization of antibody aggregation: Role of buried, unpaired cysteines in particle formation. J Pharm Sci 2010; 99:764-81.
2. Gavel Y, von Heijne G. Sequence differences between glycosylated and non-glycosylated Asn-X-Thr/Ser acceptor sites: implications for protein engineering. Protein Eng 1990; 3:433-42.
3. Jarasch A, Koll H, Regula JT, Bader M, Papadimitriou A, Kettenberger H. Developability Assessment During the Selection of Novel Therapeutic Antibodies. J Pharm Sci 2015; 104:1885-98.
4. Sydow JF, Lipsmeier F, Larraillet V, Hilger M, Mautz B, Mølhøj M, Kuentzer J, Klostermann S, Schoch J, Voelger HR, et al. Structure-Based Prediction of Asparagine and Aspartate Degradation Sites in Antibody Variable Regions. PLoS One 2014; 9:e100736-e.
5. Liu YD, Goetze AM, Bass RB, Flynn GC. N-terminal Glutamate to Pyroglutamate Conversion in Vivo for Human IgG2 Antibodies. J Biol Chem 2011; 286:11211-7.
6. Ruoslahti E. RGD AND OTHER RECOGNITION SEQUENCES FOR INTEGRINS. Ann Rev Cell Dev Bi 1996; 12:697-715.
7. Vlasak J, Ionescu R. Fragmentation of monoclonal antibodies. mAbs 2011; 3:253-63.
8. Almagro JC, Teplyakov A, Luo J, Sweet RW, Kodangattil S, Hernandez-Guzman F, Gilliland GL. Second antibody modeling assessment (AMA-II). Proteins 2014; 82:1553-62.
9. North B, Lehmann A, Dunbrack RL. A New Clustering of Antibody CDR Loop Conformations. J Mol Biol 2011; 406:228-56.
10. Shirai H, Ikeda K, Yamashita K, Tsuchiya Y, Sarmiento J, Liang S, Morokata T, Mizuguchi K, Higo J, Standley DM, et al. High-resolution modeling of antibody structures by a combination of bioinformatics, expert knowledge, and molecular simulations. Proteins 2014; 82:1624-35.
11. Weitzner BD, Kuroda D, Marze N, Xu J, Gray JJ. Blind prediction performance of RosettaAntibody 3.0: Grafting, relaxation, kinematic loop modeling, and full CDR optimization. Proteins 2014; 82:1611-23.
12. Marcatili P, Olimpieri PP, Chailyan A, Tramontano A. Antibody structural modeling with prediction of immunoglobulin structure (PIGS). Nat Protocols 2014; 9:2771-83.
13. Biasini M. pv: v1.8.1. Zenodo. doi:10.5281/zenodo.2620 2015.
